# Urobilin Test in Malaria

**Published:** 1915-11

**Authors:** 


**Urobilin Test in Malaria.** J. R. Snyder, Sacramento, Cal. State Jour. of Med., July. Urobilin is formed in the intestine by reduction of bilirubin through bacterial activity. Normally, it is re-excreted as bilirubin but in any inflammation (or other serious lesion) of the liver, it reaches the blood as such and a part is excreted in the urine. The urine is treated with an equal amount of saturated solution of zinc acetate in absolute alcohol. To about 5 c.c. of such a mixture, 2 drops of Lugol's solution (Iodine 2%, KI 4% in water) are added. Filter. If urobilin is present, the filtrate is fluorescent. The test is particularly diagnostic for malaria. Of 56 malarial cases by clinical diagnosis, 14 gave positive blood findings, 46 showed urobilin. In 40 others, there were only 3 positive tests, 2 late cases of pulmonary tuberculosis and one of cancer of the liver with cachexia.

